# Taxonomic and nomenclatural notes on *Pedicularis* (Orobanchaceae): I. One new species from northwest Yunnan, China

**DOI:** 10.3897/phytokeys.130.35258

**Published:** 2019-08-29

**Authors:** Xin Li, Hong Wang, De-Zhu Li, Wen-Bin Yu

**Affiliations:** 1 Center for Integrative Conservation, Xishuangbanna Tropical Botanical Garden, Chinese Academy of Sciences, Mengla, Yunnan 666303, China; 2 University of Chinese Academy of Sciences, Shijingshan District, Beijing 100049, China; 3 Key Laboratory for Plant Diversity and Biogeography of East Asia, Kunming Institute of Botany, Chinese Academy of Sciences, Kunming, Yunnan 650201, China; 4 Plant Germplasm and Genomics Center, Germplasm Bank of Wild Species, Kunming Institute of Botany, Chinese Academy of Sciences, Kunming, Yunnan 650201, China; 5 Southeast Asia Biodiversity Research Institute, Chinese Academy of Science, Yezin, Nay Pyi Taw 05282, Myanmar

**Keywords:** Orobanchaceae, *Pedicularis multicaulis*, Mountains of Southwest China, phylogenetic analysis

## Abstract

*Pedicularis multicaulis* W.B.Yu, H.Wang & D.Z.Li (series *Oliganthae* Prain) is a new species described and illustrated herein. This new species is endemic to northwest Yunnan and only two populations were found in Weixi county. Phylogenetic analyses support *P. multicaulis* as a new species, sister to *P. taihaiensis* Bonati and *P. macilenta* Franch. Morphological comparisons between *P. multicaulis* and *P. macilenta* and *P. taihaiensis* also support *P. multicaulis* as a new species to science.

## Introduction

*Pedicularis* Linn., with around 600 species, is the largest genus of Orobanchaceae and widely distributed throughout the North temperate region (Fischer 2004, Stevens 2001, Yu et al. 2015). More than 350 species have been recognised in China (Yang et al. 1998). Of them, about two-thirds of the species are restricted in the Hengduan Mountains, which belongs to the Mountains of Southwest China hotspot (Wang 2006, Wang and Wu 1994). Due to the previously limited accessibility of the Mountains of Southwest China before the 21^st^ century, several new species of *Pedicularis* have subsequently been discovered and described in the 2000s, owing to the construction of a road system under China’s Great Western Development Strategy (Liu and Yu 2015, Yang et al. 2003, Yu et al. 2010, Yu et al. 2018).

According to the phylogeny of the *Pedicularis* species with well-represented samples from the Hengduan Mountains region, 18 taxa were not categorised as any recognised species, based on both molecular and morphological data (Yu et al. 2015), which could be potential new species or new records to China. Of them, two taxa had been described as new species, *P. wanghongiae* M.L.Liu & W.B.Yu (Liu and Yu 2015) and *P. millina* W.B.Yu, D.Z.Li & H.Wang (Yu et al. 2018). In this study, we described and illustrated another new species, *P. multicaulis* W.B.Yu, H.Wang & D.Z.Li, from the remaining 16 taxa after carefully examining morphological characters and in comparisons with herbarium specimens of the close relatives, *P. taihaiensis* Bonati and *P. macilenta* Franch. (Yu et al. 2015). *Pedicularis multicaulis* is strongly supported as a new species, based on the revised phylogenetic analyses. Meanwhile, the pollen morphology of *P. multicaulis* was investigated using a scanning electron microscope (SEM).

## Material and methods

The fresh specimens of the new species were collected from Pantiange and Lidiping in Weixi county, northwest Yunnan, China. Pollen samples were collected from the type specimens, then observed under SEM (ZEISS EVO LS10, Germany). For the morphological comparisons, we examined specimens or specimen images of the closest relatives from the herbaria E, K, KUN, LA, P and PH. Selected type specimens of *P. macilenta* and *P. taihaiensis* are presented in Suppl. material 1: Figures S1 and S2.

According to the published phylogeny of *Pedicularis* (Yu et al. 2015), *P. multicaulis*, *P. macilenta* and *P. taihaiensis* were chosen as ingroups and *P. cephalantha* Franch. ex Maxim. and other species from series *Oliganthae* Prain, *Strobilaceae* Tsoong and *Amplitubae* Li were also included (Table 1). *Pedicularis axillaris* Franch. ex Maxim. was specified as the outgroup. In this study, we had two samples of the new species from Pantiange (*W.-B. Yu et al. 2014102*) and Lidiping (*W.-B. Yu et al. 2014096*), respectively, two samples of *P. tahaiensis* from Luquan (*C.-L. Xiang et al. HP9544*) and Huize (*W.-B. Yu et al. HW10369*), respectively and one sample of *P. macilenta*. Four DNA regions (nrITS, *matK*, *rbcL* and *trnL-F*) were used and the new sequences generated following Yu et al. (2011). Bayesian Inference (BI), Maximum Likelihood (ML) and Maximum Parsimony (MP) methods were used to reconstruct the phylogenies. The BI analysis was performed using MrBayes 3.26 (Ronquist and Huelsenbeck 2003). The total dataset was partitioned (see Suppl. material 2: Dataset 1) and the DNA substitution model of Bayesian Information Criterion (BIC) for four DNA regions was estimated using jModeltest 2 (Darriba et al. 2012). The ML analysis was conducted with RAxML 8.2.10 (Stamatakis et al. 2008). The MP analysis was carried out using PAUP* 4.a165 (Swofford 2003). Parameters for the three analyses followed the previous studies (Yu et al. 2013, Yu et al. 2015).

**Table 1.** Voucher information and GenBank accessions of samples used in phylogenetic analyses.

**Taxon**	**Source**	**Voucher information**	**ITS**	***matK***	***rbcL***	***trnL-F***
*P. amplituba* H.L. Li	Yunnan: Luquan	Yu et al., LIDZ1519A (KUN)	JF977469	JF955063	JF942952	KF277605
*P. axillaris* Franch. ex Maxim. (3)	Yunnan: Dali	Yu et al., YWB2014097 (KUN)	KT022428	KT022531	KT022705	KT022883
*P. cephalantha* Franch. ex Maxim.	Yunnan: Lijiang	W. Jiang, 08727 (KUN)	JF977493	JF955087	JF942976	KF277613
*P. cephalantha* affinis	Yunnan: Eryuan	Yu et al., YWB2014063	KT022501	KT022661	KT022841	KT022967
*P. dissectifolia* H.L. Li	Yunnan: Shangeri-La	Yu et al., HW10133 (KUN)	KF277539	KR707763	KF277641	KF277641
*P. fengii* H.L. Li (1)	Yunnan: Shangeri-La	Yu et al., HW10102 (KUN)	JF977553	JF955146	JF943036	KT022910
*P. fengii* H.L. Li (2)	Yunnan: Shangeri-La	Yu et al., Yu606 (KUN)	JF977564	JF955157	JF943047	KF277646
*P. gracilicaulis* H.L. Li	Xizang: Chayu	Jin et al., STET0522 (PE)	KF277547	no data	no data	KF277654
*P. macilenta* Franch. ex Forbes ex Hemsl.	Yunnan: Zhaotong	Li et al., 8484 (KUN)	KF277558	KT022606	KT022780	KF277680
*P. multicaulis* W.B.Yu, H.Wang & D.Z.Li	Yunnan: Weixi	Yu et al., YWB2014096	KT022502	KT022662	KT022842	KT022968
*P. multicaulis* W.B.Yu, H.Wang & D.Z.Li	Yunnan: Weixi	Yu et al., YWB2014102	MK983380	MK983381	MK983382	MK983383
*P. strobilacea* Franch.	Yunnan: Shangeri-La	Cai et al., 11CS3261 (KUN)	KT022508	KT022673	KT022852	KT022977
*P. pseudocephalantha* Franch.	Xizang: Linzhi	Gao et al., GLM123906 (KUN)	KR707794	KR707760	KR707780	KR707807
*P. tachanensis* Bonati	Sichuan: Mianning	Yu et al., LIDZ1062 (KUN)	JF977743	JF955333	JF943226	KF277740
*P. tahaiensis* Bonati	Yunnan: Luquan	Xiang et al., HP9544 (KUN)	JF977552	JF955145	JF943035	KF277741
*P. tahaiensis* Bonati	Yunnan: Huize	Yu et al., HW10369 (KUN)	JF977563	JF955156	JF943046	no data

The conservation status of *P. multicaulis* was assessed in accordance with IUCN Red List Criteria (IUCN 2012).

## Taxonomy

### Pedicularis multicaulis

Taxon classificationPlantaeLamialesOrobanchaceae

W.B.Yu, H.Wang & D.Z.Li sp. nov.

CB89FC88945E56BB8D4B74B70DE62BFE

urn:lsid:ipni.org:names:77201400-1

**Figure 1.**
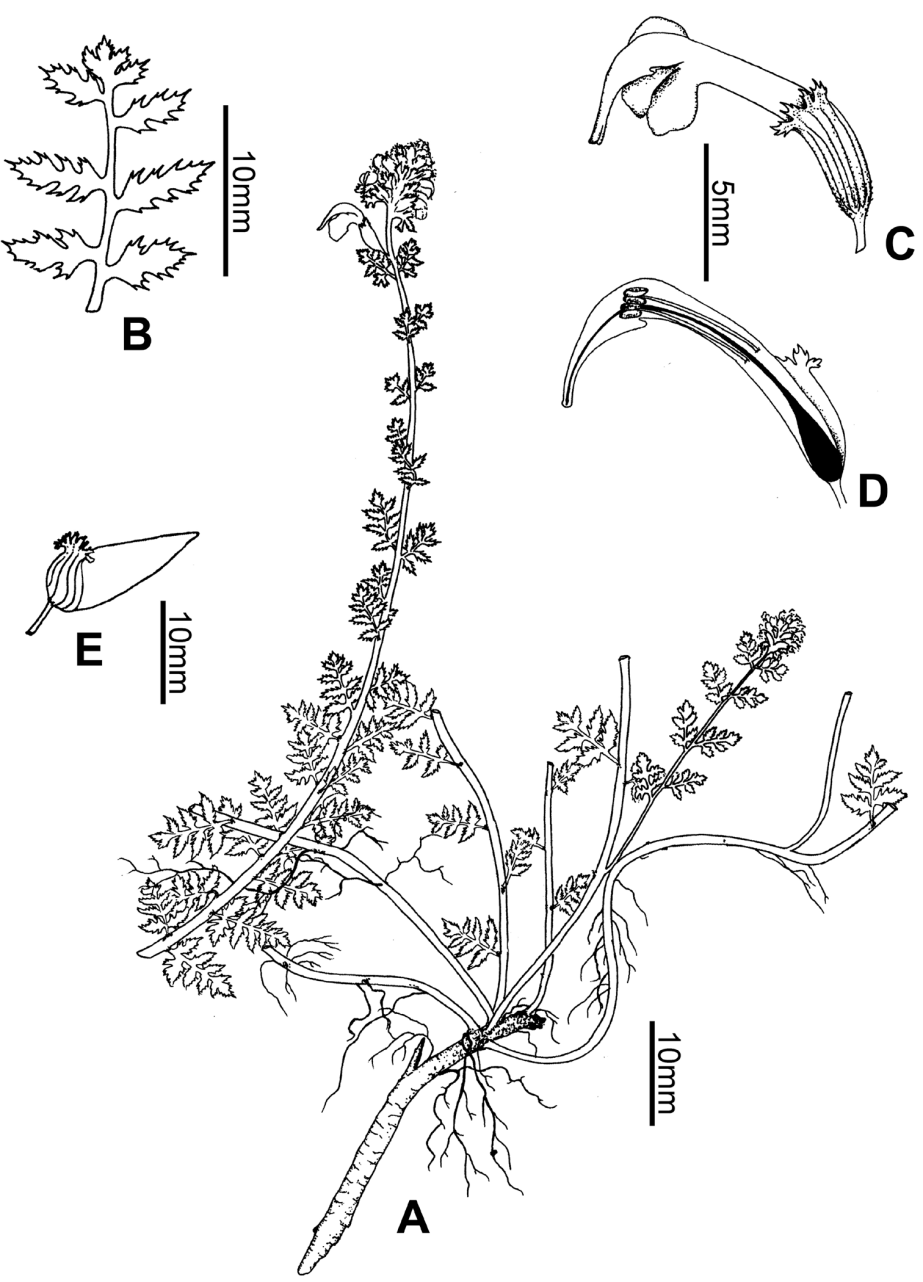
Line drawing of *Pedicularis multicaulis* W.B.Yu, H.Wang & D.Z.Li **A** habit **B** leaf **C** flower **D** open flower showing the anthers and style **E** fruit. Drawn by Zhen-Long Liang from the holotype (**A–D**) and an isotype (**E**), *W.-B. Yu, X.-L. Yang & H. Tang 2014096* (KUN).

**Figure 2.**
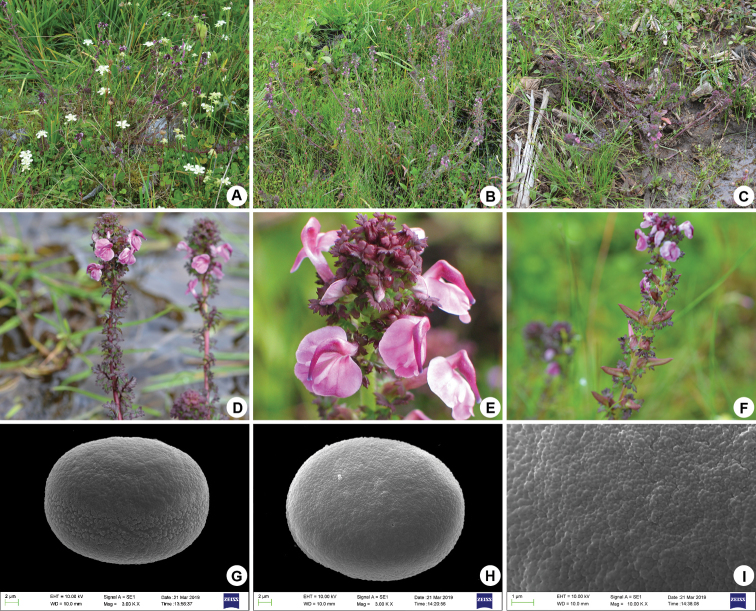
Field photos and pollen of *Pedicularis multicaulis* W.B.Yu, H.Wang & D.Z.Li **A–C** overview of habitat and plants **D** inflorescence **E** flowers **F** infructescence **G** equatorial view of pollen **H** polar view of pollen **I** exine ornamentation.

**Figure 3.**
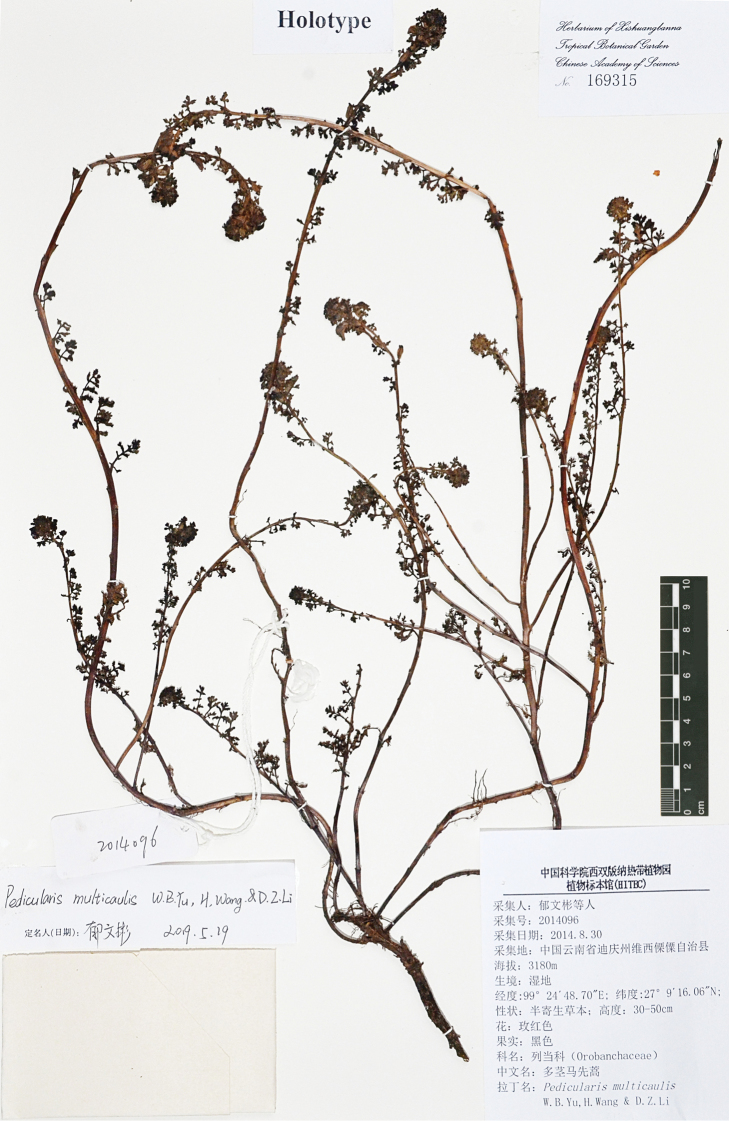
The holotype of *Pedicularis multicaulis* W.B.Yu, H.Wang & D.Z.Li (*W.-B. Yu, X.-L. Yang & H. Tang 2014096*, HITBC, accession no. 169315).

Figures 1 , 2A–F and 3

#### Vernacular name.

Duo Jing Ma Xian Hao (多茎马先蒿) (Chinese).

#### Type.

CHINA. Yunnan: Weixi, Lidiping, wet meadow, alt. 3180 m, 27°9'16.06"N, 99°24'48.70"E, 30 Aug 2014, *W.-B. Yu, X.-L. Yang & H. Tang 2014096* (holotype: HITBC! (accession no. 169315); isotypes: HITBC!, KUN!).

#### Diagnosis.

*Pedicularis multicaulis* W.B.Yu, H.Wang & D.Z.Li is distinguished from *P. macilenta* and *P. taihaiensis* in having taller and more ascending stems, partially crawling stems with fibrous roots, shorter petiole and leaf blade of cauline leaves in middle and upper parts and smaller corollas with a shorter beak.

#### Description.

Herbs perennial, 20–50 cm tall, glabrescent, drying slightly black; taproots slender, fusiform; stems caespitose, mostly (3) 5 to 9 (12) from a caudex, ascending or partially crawling (with fibrous roots) and branchlets (0) 1–3 (10), glabrescent or sparely pubescent along the lines. Basal leaves absent. Cauline leaves alternate; petiole up to 10 mm long or distal ones sessile or subsessile, glabrescent; leaf blade ovate-elliptic or oblong, 5–30 mm × 7–15 mm, glabrous on both surfaces, pinnatisect; segments 2 to 5 pairs, ovate to lanceolate-oblong, incised-pinnatifid or double dentate. Inflorescences racemose, up to 30 cm long; bracts leaflike, distal ones shorter than flowers. Pedicel 1.0–2.5 mm long. Calyx tube ca. 5 mm long, glabrescent, 1/3 cleft anteriorly; lobes 3, unequal, posterior one acicular, lateral pair larger, leaf-like and toothed. Corolla rose, 10–14 mm long; tube erect, ca. 8–10 mm long ; galea ±falcate, not crested, not twisted, with 1 distinct reflexed marginal tooth on one side; beak straight, ca. 3 mm, slightly 2-cleft at apex, not ciliate; lower lip 5–6 mm × 6–8 mm, sparely ciliate, lobes 3 unequal; middle lobes apex slightly cucullate. Filaments 4 glabrous, equal length, ca.13 mm long, inserted in the middle of corolla. Ovary long ovoid, ca. 3 mm long; Capsule lanceolate-oblong, 10–15 mm × 4–5 mm. Seeds narrowly ovoid, ca. 1.0–1.2 mm.

#### Etymology.

The specific epithet “*multicaulis*” refers to the new species having many ascending stems that are branched in the middle and upper parts.

#### Phenology.

This new species was found in flowering from middle June (in a field trip in 2006) to August and in fruiting from July to September.

#### Pollen morphology.

Pollen grains are radially symmetrical, isopolar, spheroidal and medium in size (polar length: 23.71–25.47 μm × equatorial diameter: 18.86–20.29 μm). Pollen apertures are bisyncolpate (Figures 2G and H) and the colpi are usually wide and sunken (Figure 2G); exine ornamentation is perforated tectum with microfoveolate ornamentation (Figure 2I).

#### Phylogenetic analyses.

**Figure 4.**
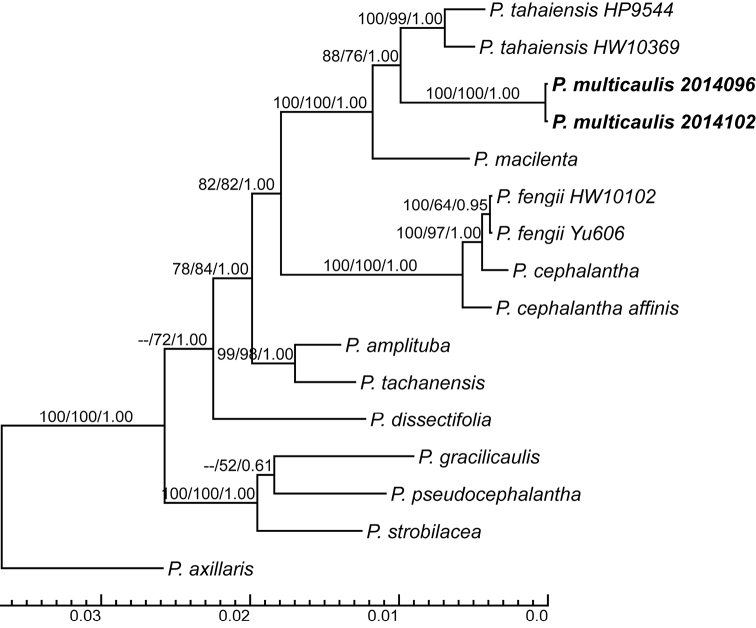
The major-rule consensus tree of Bayesian Inference analysis using the total data by concatenating four DNA regions (nrITS, *matK*, *rbcL* and *trnL-F*). Bootstrap values of Maximum Likelihood/Parsimony and posterior probability values of Bayesian Inference are presented above branches. The bottom scale bar represents the number of substitutions per site.

All analyses strongly supported *P. taihaiensis* as sister to *P. multicaulis* (ML/MP/BI = 88/76/1.00, Figure 4) and the two samples of *P. multicaulis*(ML/MP/BI = 100/100/1.00) and of *P. taihaiensis* (ML/MP/BI = 100/99/1.00) are monophyletic, respectively. Then, *P. macilenta* is sister to *P. taihaiensis* + *P. multicaulis* (ML/MP/BI = 100/100/1.00).

#### Distribution.

**Figure 5.**
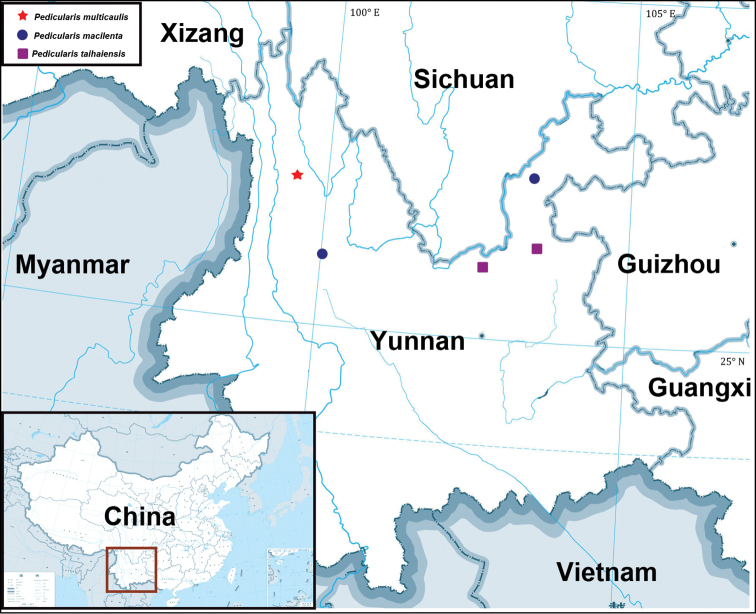
Distribution map of *Pedicularis multicaulis* W.B.Yu, H.Wang & D.Z.Li and related taxa.

*Pedicularis multicaulis* was only found in two populations in Weixi county, northwest Yunnan (Figure 5). It occurs in wet meadow or the margin of wetland between 2900 m and 3200 m a.s.l.

#### Conservation assessment.

To date, we only collected this new species from two populations in Weixi county, northwest Yunnan. There are around 100 and 300 individuals in Pantiange and in Lidiping, respectively. It is restricted to wet meadow, which is likely to be threatened by grazing in these areas. According to IUCN Red List Criteria (IUCN 2012), *P. multicaulis* can be classified as Vulnerable (VU).

#### Additional examined specimens.

*Pedicularis multicaulis* W.B.Yu, H.Wang & D.Z.Li. CHINA. Yunnan: Weixi, Pantiange, wet grassland, alt. 2930 m, 27°20'39.48"N, 99°16'59.30"E, 27 Aug 2014, *W.-B.Yu, X.-L.Yang & H.Tang 2014102* (KUN!). *Pedicularis macilenta* Franch. CHINA. Yunnan: Eryuan (Mountain Yentzehay), in humid localities on the slopes, 8 Aug 1888, *Delavay 3698* (types, P!, PH!, LA!); Yunnan: Zhaotong, Dashanbao, Dahaizi reservoir, alt. 3044 m, 27°44'89.2"N, 103°31'94"E, 7 Aug 2008, *H.Li et al. 8078* (KUN!). *Pedicularis taihaiensis* Bonati. CHINA. Yunnan: Huize, Dahai, Jul 1913, *E.E. Maire 678* (holotype: E [E00284020]!); ibid. 30 Jul 2010, *W.-B.Yu et al. HW10369* (KUN!); Yunnan: Luquan, Wumeng Mountains, alt. 3700 m, 2 Jul 1990, *R.Z.Fan & Z.W.Lyv 061* (KUN!); Yunnan: Luquan, Jiaozi Mountain. 8 Jul 2008, *C.L.Xiang et al. HP9544* (KUN!).

## Discussion

The galea of *P. multicaulis* bears one pair of distinct reflexed marginal teeth on both sides, which is the key character of series *Oliganthae* Prain. Phylogenetic analyses did not support series *Oliganthae* as monophyletic (Yu et al. 2015). The previous study indicated that *P. macilenta* and *P. taihaiensis* formed a weakly supported clade, then sister to *P. multicaulis* (= *Pedicularis* sp. (9)) by using one sample of each species. In this study, both *P. multicaulis* and *P. taihaiensis* had two samples from different populations and our results showed that *P. multicaulis* and *P. taihaiensis* formed a strongly supported clade, then sister to *P. macilenta*. The relationship amongst the three species was well resolved. Therefore, population level sampling is very important for species delimitation and phylogeny of recently derived lineage.

Morphological characters differentiate *P. multicaulis* from the two most closely related species (Table 2). The key diagnostic characters of *P. multicaulis* are having taller and more branched stems, partially crawling stems with fibrous roots, shorter petiole of cauline leaves and smaller corollas with a short beak. The three species are also isolated geographically (Figure 5). According to herbarium records, *P. taihaiensis* occurs in Luquan and Huize, north Yunnan and *P. multicaulis* is only found in Weixi, northwest Yunnan. The distribution of *P. macilenta* is disjunct, with one population in Eryuan, northwest Yunnan and another in Zhaotong, northeast Yunnan. As all three species were mainly confined to the habitat of wet meadow, we assume that geographical isolation may play an important role in species divergence in this lineage.

**Table 2.** Morphological comparison amongst *Pedicularis multicaulis*, *P. macilenta* and *P. taihaiensis*.

**Characters**	***P. multicaulis***	***P. macilenta***	***P. taihaiensis***
Plant height (cm)	20–50	20–30	15–30
Rooting stems	Yes	No	No
Stems	(3) 5-9 (12)	1–5	2–4
Branchlets per stem	(0) 1-3 (10)	1–3	1–3
Leaf bade size (mm)	5–20 × 7–15	30–50 × 10–15	15–30 × 8–11
Petiole length (mm)	3–11	5–20	8–25
Leaf lobes (pairs)	2–5	5–7	5–7
Leaf lobe size (mm)	3–8 × 2–4	3–7 × 2–5	3–6 × 1–4
Calyx length (mm)	4–5	6–7	5–7
Corolla colour	Rose	White with purple beak	Rose
Corolla length (mm)	10–14	11–13	17–20
Corolla tube length (mm)	8–10	6–7	11–15
Beak length (mm)	3	3–4	4–5
Galea	Not crested	Slightly crested	Not crested

## Supplementary Material

XML Treatment for Pedicularis multicaulis

## Supplementary materials

Supplementary material 1 Figures S1 and S2 Data type: Specimen photos. Explanation note: Figure S1. A syntype of *Pedicularis macilenta* Franch. (Delavay 3698, P [P02981467]); Figure S2. The holotype of *Pedicularis taihaiensis* Bonati (E.E. Maire 678, E [E00284020]). https://binary.pensoft.net/file/327944This dataset is made available under the Open Database License (http://opendatacommons.org/licenses/odbl/1.0/). The Open Database License (ODbL) is a license agreement intended to allow users to freely share, modify, and use this Dataset while maintaining this same freedom for others, provided that the original source and author(s) are credited.Xin Li, Hong Wang, De-Zhu Li, Wen-Bin Yu

Supplementary material 2 Dataset 1 Data type: DNA matrix Explanation note: DNA sequence matrix of the four DNA markers with the best-fit BIC models . The matrix is partitioned by regions. https://binary.pensoft.net/file/327945This dataset is made available under the Open Database License (http://opendatacommons.org/licenses/odbl/1.0/). The Open Database License (ODbL) is a license agreement intended to allow users to freely share, modify, and use this Dataset while maintaining this same freedom for others, provided that the original source and author(s) are credited.Xin Li, Hong Wang, De-Zhu Li, Wen-Bin Yu
